# Metal-Organic Frameworks-Based Optical Nanosensors for Analytical and Bioanalytical Applications

**DOI:** 10.3390/bios13010128

**Published:** 2023-01-12

**Authors:** Cong Wen, Rongsheng Li, Xiaoxia Chang, Na Li

**Affiliations:** 1 Beijing National Laboratory for Molecular Sciences (BNLMS), Key Laboratory of Bioorganic Chemistry and Molecular Engineering of Ministry of Education, College of Chemistry and Molecular Engineering, Peking University, Beijing 100871, China; 2 National Demonstration Center for Experimental Chemistry and Chemical Engineering Education (Yunnan University), School of Chemical Science and Engineering, Yunnan University, Kunming 650091, China; 3 College of Chemistry and Molecular Engineering, Peking University, Beijing 100871, China

**Keywords:** metal–organic frameworks, luminescence, surface-enhanced Raman spectroscopy, multiplexed detection

## Abstract

Metal-organic frameworks (MOFs)-based optical nanoprobes for luminescence and surface-enhanced Raman spectroscopy (SERS) applications have been receiving tremendous attention. Every element in the MOF structure, including the metal nodes, the organic linkers, and the guest molecules, can be used as a source to build single/multi-emission signals for the intended analytical purposes. For SERS applications, the MOF can not only be used directly as a SERS substrate, but can also improve the stability and reproducibility of the metal-based substrates. Additionally, the porosity and large specific surface area give MOF a sieving effect and target molecule enrichment ability, both of which are helpful for improving detection selectivity and sensitivity. This mini-review summarizes the advances of MOF-based optical detection methods, including luminescence and SERS, and also provides perspectives on future efforts.

## 1. Introduction

Nanomaterial-based optical methods are advantageous for measuring quantitatively biomolecules of interest. With optical nanosensors, an extremely high photoluminescence intensity or enhancement effect in surface-enhanced Raman spectroscopy (SERS) can be achieved, and diversified signals, including wavelength/wavenumber, intensity, and excited-state lifetime, can be tuned to cater to specific applications. Most importantly, multiple signals can be integrated in a single optical nanoprobe to readily achieve ratiometric, multiplexed, and multimodality measurements. These detection modes are highly desirable for quantitative measurements in complex sample matrices [1,2].

Metal–organic frameworks (MOFs) are a kind of hybrid porous material consisting of inorganic metal ion or cluster nodes, and linkers including organic ligands and metal–organic complexes [3,4,5,6,7]. Due to the large specific surface area, the ultrahigh porosity, the adjustable internal surface property, the extraordinarily diversified structure, and the reasonable biocompatibility, MOFs are widely employed in storage and separation [8], catalysis, [9,10] drug delivery [5,11,12,13,14,15], and biomedicine [12,16,17,18], as well as in chemical sensors and biosensors [12,19,20,21,22,23,24,25,26,27,28]. In terms of optical measurements, MOFs are promising optical sensing materials because emission centers of MOFs can be constructed by “multiple photonic units” originating from inorganic metal ion or cluster nodes, linkers, or their combination to exemplify the features of structural diversity through combining inorganic and organic chemistry [29]. This unique property, together with tunable functional sites, imparts MOFs with highly designable and diversified luminescence that can be used for customized applications. On one hand, the “multiple photonic units” can be engineered or tailored with rational design to achieve the aforementioned diversified luminescence signals, all of which can be used for applications with specific needs; on the other hand, aside from attaching recognition entities for target-specific interaction, the functional sites can also be used to provide auxiliary interaction with emission centers to further tailor luminescence properties. Furthermore, taking the advantage of ultra-high porosity and modifiable internal surface property to encapsulate luminescent guest molecules in the porous structure, one more dimension of luminescence can be added to grant MOFs with ratiometric, multiplexing, and multimodality measurement capabilities [30,31]. In addition to luminescence, MOFs can serve as promising materials for SERS methods in which MOFs either act as substrates for signal enhancement or form composite substrates by encapsulating metallic nanomaterials. In addition to the above, the high surface area and controllable pore size allow a high capacity for adsorbing and concentrating analytes to achieve a low limit of detection (LOD) and a unique sieve effect, thus improving the selectivity [6,32].

There are quite a few review articles on MOFs and their applications in the optical measurement field [33,34,35]. This mini-review tries to highlight MOFs-based optical nanosensors, particularly luminescence and SERS, for applications in analytical chemistry. First, the application of MOFs in luminescence detection is summarized and discussed based on the evolution from a single luminophore, including MOFs itself or the encapsulated guest, to multiple signal sources for ratiometric and multimodality measurements. Second, the application of MOFs in SERS measurements is summarized and discussed based on the MOF itself as both a SERS substrate and a MOF-metal nanomaterial composite substrate. Finally, perspectives on future efforts to develop MOF-based nanosensors for analytical and bioanalytical applications are provided.

## 2. MOFs-Based Nanosensors for Luminescence Applications

Luminescence sensing has been recognized as an important tool in food safety detection, disease diagnosis, and environmental monitoring [36,37]. For these applications, it is required that the method has sufficient sensitivity and selectivity towards the analyte of interest. For analysis of targets in complex sample matrices, it is also desirable that the sensor or nanosensor system can self-calibrate to provide reproducible and quantitative results (Table 1).

Compared to conventional organic and inorganic luminophores, the luminescent MOF materials have excellent host and sensing features that can meet the aforementioned requirements for luminescence sensing [32,35,38]. The photoluminescence of MOFs may originate from metal centers, linkers, or guest molecules. Each emission unit can be designed and tuned for diversified application needs. Moreover, the integration of emissive units into MOFs can be adopted for the purpose of developing multi-emissive probes to realize self-calibrating measurements, multiplexed measurements, and multimodality measurements. The following discussion is based on the signal evolution in terms of the single luminophore signal, the ratiometric signal, and the multimodality signals. A perspective on multiplexed measurements for fluorescence applications will be discussed in the Conclusions and Future Perspectives section.

### 2.1. Single Luminophore Signal

The luminescence of MOFs may originate from the metal nodes [39], organic linkers [6], and guest molecules [40]. The luminescence may also originate from second building units (SBUs), including metal clusters [30,41,42] and metal–organic complex linkers [6,43], which are also used to build MOFs with extended porous networks and luminescence centers. All of the above can be utilized as luminescent probes to indicate the presence and even quantity of a tentative analyte [44]. The analyte can alter the luminescence of MOF sensors through one of the following mechanisms: intermolecular charge transfer, ligand-to-metal charge transfer (LMCT), photo-induced electron transfer (PET), Förster resonance energy transfer (FRET), dynamic quenching, or static quenching [45,46].

Lanthanide-based MOFs (Ln-MOFs), as metal node-based luminescence MOFs, present distinctive luminescence properties originating from abundant f-orbital configurations, long luminescence lifetimes, and “antenna effects” of linkers [47,48]. Qian and colleagues [39] used H_3_TATAB (4,4′,4″-s-triazine-1,3,5-triyltri-*p*-aminobenzoic acid) as an organic linker to synthesize a series of isomorphic Ln-MOFs. For example, TbTATAB exhibited good stability in water and a high fluorescence quantum yield (77.48%). The large amount of N atoms afforded it a high ligand-Hg^2+^ affinity, which was able to block the antenna effect of the linker. As a result, the fluorescence of TbTATAB was quenched through either a dynamic or a static mechanism. The TbTATAB-based sensor showed excellent stability, reproducibility, and sensitivity, such that it could be practically employed to detect Hg^2+^ in environmental samples.

**Figure 1:**
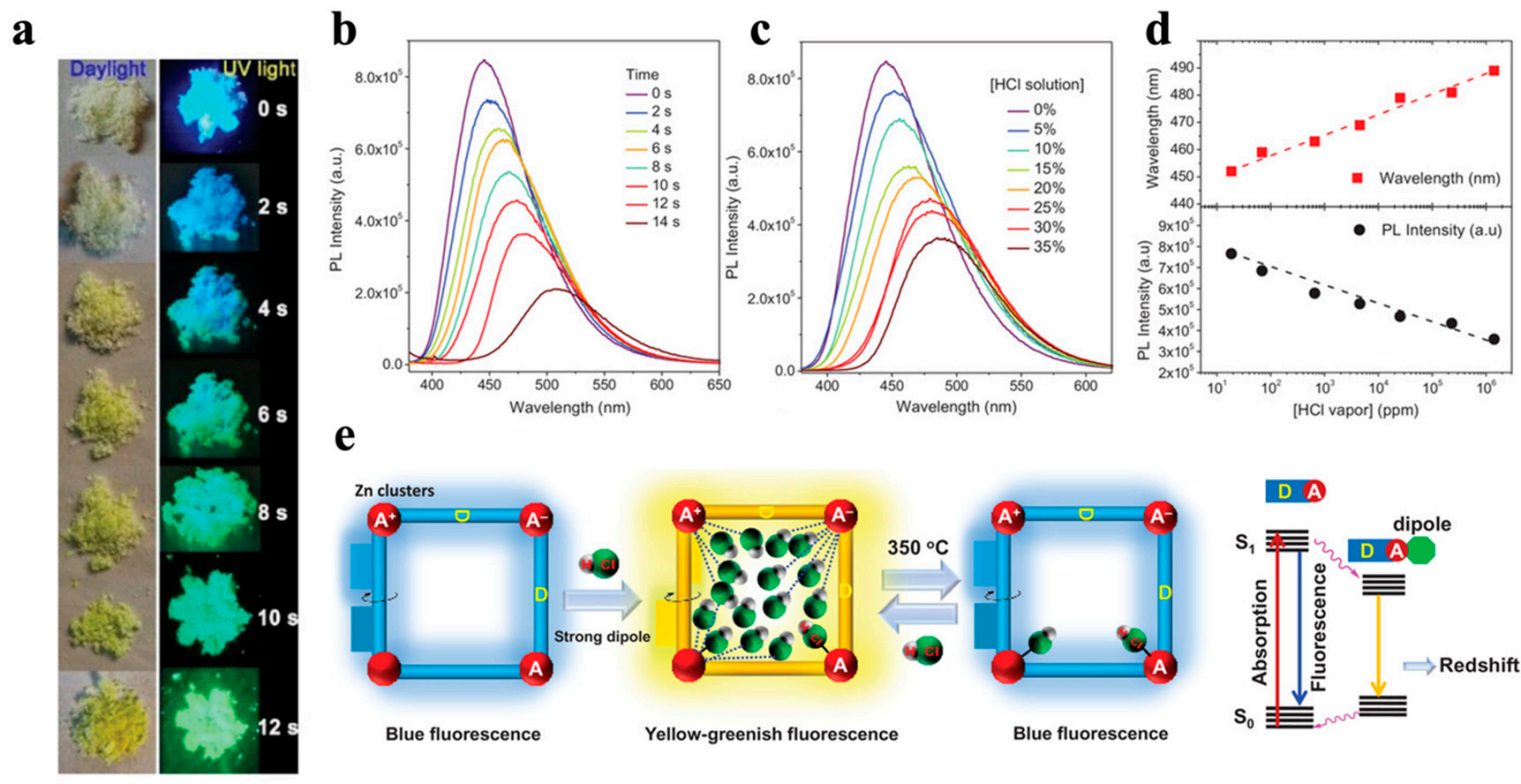
HCl vapor sensing by ZnMOF. (**a**) Photographs of ZnMOF fumigated with HCl vapor from 12% HCl solution. (**b**) Fumigation time dependence. (**c**) HCl solution concentration dependence (fumigation time = 5 s) of solid-state fluorescence spectra of HCl-vapor fumigated ZnMOF. (**d**) Emission peak and intensity responses to HCl vapor concentration. (**e**) Schematics showing the solid-state fluorescence change of ZnMOF with adsorption and desorption of HCl vapor (the blue dotted line for host–guest interaction, and the black solid line for coordination), and the energy diagram of ZnMOF showing the influence of dipole–dipole interaction [6]. Copyright 2021, John Wiley and Sons.

In addition to the metal nodes, the organic linker is another source of luminescence of MOFs. A luminescent MOF using aggregation-induced emission (AIE) molecules as an organic linker can confine the intramolecular motions of the linker molecule to give a bright emission [49]. Tang and colleagues [6] used an AIE linker, tetrakis(4-carboxyphenyl)ethylene (TCPE), to prepare ZnMOF and CoMOF with an unique [M^+^–L–M^−^–L–M]_∞_ (M = metal clusters, L = linker) configuration (Figure 1). During the sensing of HCl vapor, ZnMOF exhibited a blue-to-yellow-greenish transition of fluorescence due to the adsorption, rather than coordination, of HCl vapor. The adsorbed strong dipole HCl molecules were able to decrease the energy of the lowest singlet excited state via dipole–dipole interaction, leading to a red-shifted and weakened emission. For CoMOF, luminescence was quenched by cobalt ions. Introducing histidine could cause the collapse of the CoMOF framework and subsequent aggregation of TCPE to recover the blue emission of the TCPE aggregate.

Guest encapsulation into MOFs offers advantages over traditional synthesis of luminescent materials, such as easiness and cost effectiveness, as well as the possibility of tuning the emission properties by selection of guest molecules. [50] To date, various luminescent molecules have been used as guests, including but not limited to Lanthanide (Ln) ions [47], quantum dots (QDs) [51,52], carbon dots (CDs) [40], upconversion nanoparticles (UCNPs) [3,11,16,53,54,55,56,57,58,59], noble metal nanoclusters [5,8,38,58,60,61,62,63,64,65,66,67,68,69,70,71,72,73,74,75,76], and organic dyes [20,77,78,79,80,81,82]. Encapsulation can prevent the aggregation-caused quenching (ACQ) of organic dyes and maintain the signal stability, the photostability, and the reasonable shelf-life of the fluorescent nanoparticles [30,38,77]. Wang and colleagues [40] reported a nanoscale complex based on CDs and MOFs (abbreviated as CDs@ZIF-8) for enhanced chemical sensing of quercetin. Quercetin can form a complex with CD in CDs@ZIF-8 via the electrostatic interaction between hydroxyl groups of quercetin and basic groups on the surface of CDs; thus, the fluorescence of CDs@ZIF-8 was quenched. Moreover, ZIF-8 endowed CDs@ZIF-8 with a high binding affinity to quercetin by π-π staking, as well as by improving the detection sensitivity and selectivity. The aforementioned characteristics of CDs@ZIF-8 guaranteed a LOD of 3.5 nM and its suitability for practical application in real samples for sensing of quercetin. In addition, Yan and colleagues [47] also used Ln ions as guest molecules to decorate MOF through post-synthetic modification in order to detect diphenyl phosphate in human urinary samples.

### 2.2. Ratiometric Signal

Although single emission-based luminescent materials have been widely used, they suffer from inherent defects, such as signal fluctuations, variations of probe concentrations, light scattering from the matrix, signal fluctuations due to the complex matrices and sometimes sample pretreatments, etc. [30,77,79,83,84,85,86]. The ratiometric fluorescence sensing, based on the intensity ratio of two or more well-resolved emission bands, has the self-calibrating capability to eliminate the aforementioned problems, and, thus, to enable more accurate measurements [80,84,87,88]. Due to the tunable multiple emission centers, the large absorption cross sections, and the tailorable skeletons [78,80], MOFs have been demonstrated to be a potential candidate for ratiometric sensing applications [78,79,83,84]. A ratiometric signal can be achieved between MOF and guest molecules [30,78,79,80,89], between guest and guest molecules [77], and even by MOF itself [32,42,84].

**Figure 2:**
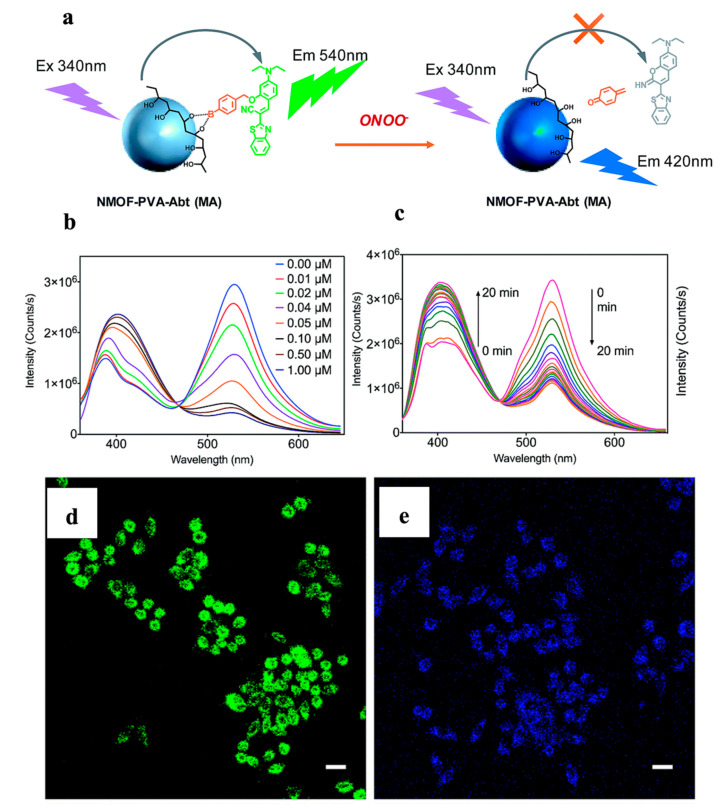
The sensing and cell imaging of ONOO^-^ with NMOF-Dye FRET pair. (**a**) Schematics of the sensing mechanism. (**b**) Fluorescence spectra of MA (1 mg L^−1^) as a function of ONOO^−^ concentration in PBS buffer (pH 7.4) for 30 min; (**c**) fluorescence spectra of MA (1 mg L^−1^) with the addition of 1.00 mM ONOO^−^ over time (0 to 20 min). (**d**,**e**) Fluorescence microscopic images of HeLa cells for exogenous ONOO^−^. (**d**) The cells were stained with MA (10 mg L^−1^) for 1 h and then washed with PBS before imaging; (**e**) the cells were pretreated as shown in (**d**) and then treated with SIN-1(1 mM) for 30 min. Scale bar: 25 mm [78]. Copyright 2017, Royal Society Chemistry.

The tailor-made skeletons of MOFs offer specific host–guest interaction sites for intended recognition events. The guest molecules can be attached on the surface of MOFs through covalent linking [78] and electrostatic adsorption [40], or being encapsulated into the MOF channels [30,38,89]. Zhang and colleagues [78] synthesized nanoscale MOF (NMOF) for ratiometric peroxynitrite (ONOO^−^) sensing based on FRET (Figure 2). Poly(vinyl alcohol) (PVA) was used to attach the energy acceptor (ABt or BDP) on the surface of the energy donor (MOF). With 340-nm excitation of the donor, the nanosensor presented the emissions of the acceptors: 540 nm for ABt and 610 nm for BDP. The presence of ONOO^−^ disabled FRET by detaching the acceptor from the donor. The quantification of ONOO^−^ was realized based on the donor-to-acceptor intensity ratio. The fast response and high selectivity made the nanosensor suitable for imaging of ONOO^−^ in living cells.

Yin and colleagues [89] reported a turn-on ratiometric fluorescent sensor, Ru@MIL-NH_2_, for water quantification. Ru(bpy)_3_^2+^ was trapped in the channels of MIL-101(Al)-NH_2_ via a simple one-pot method. With the water content increasing from 0% to 100%, MIL-NH_2_ emission at 465 nm was intensified while the Ru(bpy)_3_^2+^ emission remained stable at 615 nm with 300 nm excitation. It was revealed that the protonation of the nitrogen atom of the MIL-NH_2_, the π-conjugation system, and the stable fluorescence of Ru(bpy)_3_^2+^ together facilitated the sensitive ratiometric measurements. This turn-on ratiometric fluorescence sensor showed low LOD (0.02%), fast response (less than 1 min), large dynamic range (0−100%), and good sensor reusability. The turn-on response is much simpler and more straightforward, and even more sensitive, than the quenching process [77].

A ratiometric signal can be achieved from the guest molecules and metal nodes of MOFs. Chen and colleagues [80] developed a MOF for real-time ratiometric fluorescent monitoring of food freshness by covalently coupling fluorescein 5-isothiocyanate (5-FITC) with NH_2_-rich EuMOF in a post-synthetic modification manner. Histamine, a biogenic amine produced by spoiled food, increased the emission of FITC at 525 nm and decreased the emission of Eu^3+^ at 611 nm. By doping the EuMOF-FITC probe on a flexible substrate (glass fiber), the complex was able to be integrated with a smartphone-based portable platform for on-site visual inspection of the freshness of raw fish samples.

**Figure 3:**
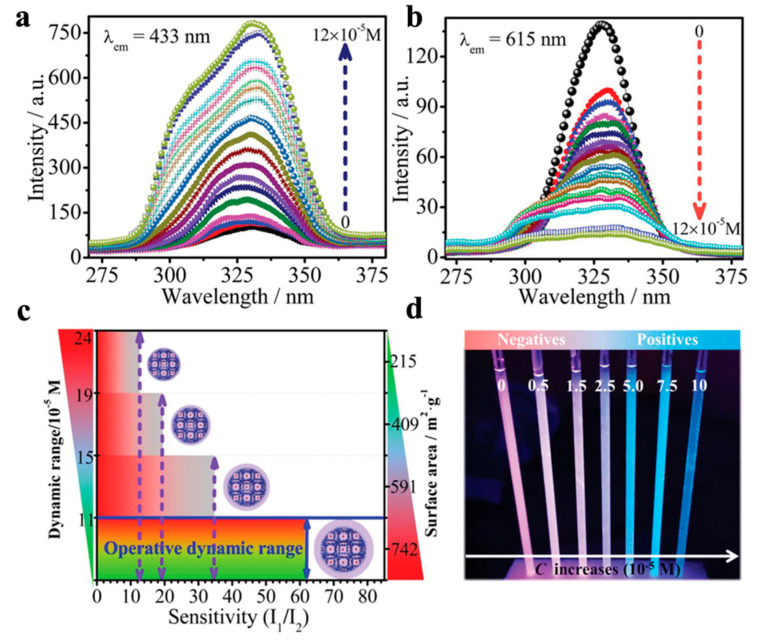
Ratiometric luminescence sensing of VMA by Eu-ZnMOF-*n*. (**a**,**b**) The widening of the excitation peak of Eu-ZnMOF-*n,* monitored at both 433 and 615 nm emissions with incremental addition of VMA. (**c**) The structure–property relationship of the probe’s surface area to its dynamic range and sensitivity. (**d**) Color-coded digital array of Eu-ZnMOF-*n* probe after treatment with increased VMA concentrations under irradiation with a UV lamp (λ_ex_ = 254 nm). [32] Copyright 2020, John Wiley and Sons.

MOFs with multi-emission centers are able to provide the ratiometric signal themselves. The metal-to-ligand and metal-to-metal energy transfers empower diversified luminescence responses. Shi and colleagues [32] synthesized a luminescent Eu-ZnMOF-*n* by a structure engineering strategy, rendering the material enhanced slope sensitivity within the “optimized useful detection window” (Figure 3). Therefore, this biosensor enabled the discrimination of small concentration variations of urinary vanillylmandelic acid (VMA), an early pathological signature of pheochromocytoma. Upon the addition of VMA, emissions from organic linker at 433 nm became conspicuous with 330-nm excitation, while emissions from Eu^3+^ at 615 nm decreased. The organic linker emission change was attributed to the formation of an exciplex between the linker and VMA, which held a lower-lying excited-state energy level; the emission change in Eu^3+^ was due to the static quenching by VMA. This structure engineering strategy provided a facile approach to detect the biomarker change within a small concentration range, making the biosensor more suitable for clinical applications.

The similar atomic radii and chemical properties of Ln ions make it feasible to prepare multi-emissive Ln-MOFs [87,90]. Previous studies have shown that a Tb-to-Eu energy transfer can occur [91]; therefore, the Tb/Eu mixed-Ln MOF can serve as the ratiometric sensor. Chen and colleagues [84] developed a mixed Ln-MOF luminescence thermometer using 2,5-dimethoxy-1,4-benzenedicarboxylate (DMBDC) as the linker. The Tb-to-Eu luminescent intensity ratio in Tb-DMBDC and Eu-DMBDC decreased as the temperature increased due to the thermal activation of non-radiative decay pathways. In the mixed-Ln-MOF, Eu_0.0069_Tb_0.9931_-DMBDC, the increment of temperature led to a decrease in Tb emissions and an increase in Eu emissions, thus forming a ratiometric fluorescence nanothermometer that presented a wide temperature dynamic range. The unique phenomenon can be attributed not only to the efficient antenna effect between DMBDC and Ln ions, but also to the Tb-to-Eu energy transfer.

Some non-luminescent MOFs, such as ZIF-8, can serve as host matrices to encapsulate guest organic dyes for ratiometric sensing. The confinement and isolation of the organic dyes can effectively inhibit the intramolecular torsional motion and increase the conformational rigidity to produce a high quantum yield [92]. Qian and colleagues [77] reported a luminescent nanothermometer by encapsulating luminescent dyes, 4-methylumbelliferone (4-Mu), and fluorescein (Flu) in the pores of ZIF-8. The developed nanothermometer can response to temperature changes based on the Flu-to-4-MU emission ratio (*I*_Flu_/*I*_4-MU_) as well as the emission peak wavelength of 4-MU. These two kinds of readouts can self-calibrate to ensure the accuracy of the detection. Furthermore, the nanosized property of ZIF-8 and the excellent luminescence properties of dyes impart the sensor with a large dynamic range and a high spatial resolution, which are important for temperature mapping [93].

**Figure 4:**
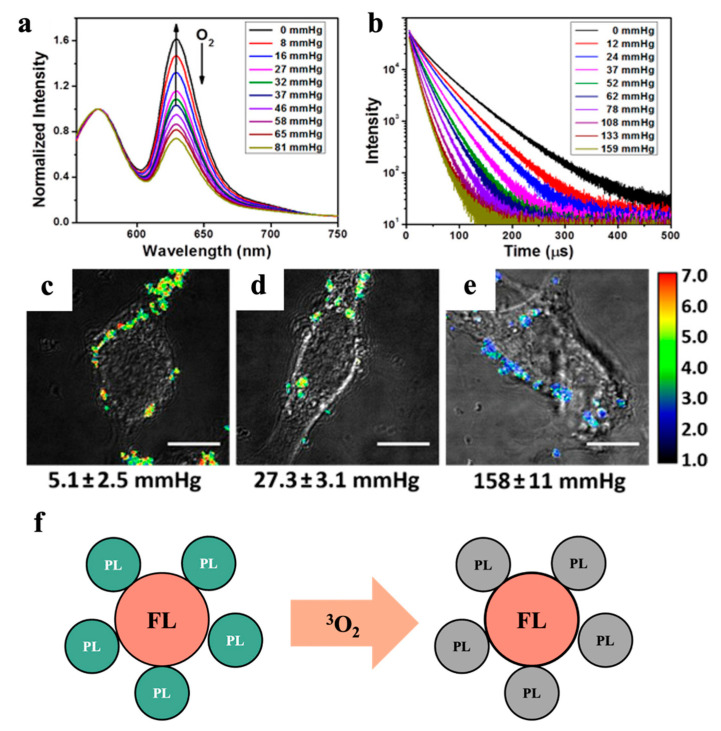
Oxygen sensing based on the phosphorescence-to-fluorescence ratio of R-UiO-1. (**a**) Emission spectra (λ_ex_ = 514 nm) and (**b**) phosphorescent decays (λ_ex_ = 405 nm) of R-UiO-1 in HBSS buffer under varied oxygen partial pressures. Ratiometric luminescence imaging (λ_ex_ = 514 nm) of CT26 cells after incubation with R-UiO-2 under (**c**) hypoxia, (**d**) normoxia, and (**e**) aerated conditions. Scale bar: 10 μm. (**f**) Schematics of fluorescence (Fl)–phosphorescence (Ph) dual emission centers, with Ph center quenched (Qu) in response to oxygen [79]. Copyright 2016, American Chemical Society.

In addition to fluorescence, phosphorescence can also be tuned by rationally designing the structure of metal nodes or organic linkers of MOFs [42,79]. MOFs can integrate both fluorescence and phosphorescence in one nanosensor. For example, the emission and lifetime of phosphorescence, but not fluorescence, are easily quenched by triplet oxygen, which can be utilized as a ratiometric signal based on the fluorescence-to-phosphorescence ratio. Lin and colleagues [79] designed the mixed-linker nanoscale UiO MOF and decorated the structure with Rhodamine-B isothiocyanate (RITC) to form R-UiO MOF for ratiometric sensing of intracellular O_2_ (Figure 4). In the nanostructure, the phosphorescent Pt-5,15-di(*p*-benzoato)porphyrin (DBP-Pt) linker acted as an O_2_-sensitive probe, and the O_2_-insensitive fluorescent RITC served as a reference. With 514-nm laser excitation, emissions at 630 nm from DBP-Pt and 570 nm from RITC were observed. With the increase of O_2_ pressure, the DBP-Pt phosphorescence decreased significantly while the RITC fluorescence remained unchanged. The intracellular O_2_ of CT26 cells at 4, 32, and 160 mmHg was detected based on ratiometric signals using confocal laser scanning microscopy.

Zang and colleagues [42] reported a fluorescence–phosphorescence dual-emissive oxygen sensing MOF: ([Ag_12_(SBu^t^)_8_(CF_3_COO)_4_(bpy-NH_2_)_4_]_n_ (abbreviated as Ag_12_bpy-NH_2_)), based on silver–chalcogenolate-cluster and bipyridine (bpy) linkers. The introduction of the amino group enhanced the spin–orbit coupling and increased the intersystem crossing efficiency to boost triplet excitons and prolong the lifetime of phosphorescence at 556 nm in vacuum. As a result, oxygen molecules quenched the phosphorescence at 556 nm, while the fluorescence emission at 456 nm remained nearly invariant. This ratiometric quantification manner ensured a LOD as low as 0.1 ppm. The introduction of other substitutional groups, such as methyl or F^−^ groups, extended the dynamic range of the ratiometric sensing. Therefore, tailoring the linker was deemed to be a powerful method for modulating luminescent sensing functionality.

Zhou and colleagues [94] used Prussian Blue (PB) and UCNPs to develop a nanoprobe (UC-PB) for the purpose of detecting and eliminating H_2_S with a linear range of 0–150 μM and an LOD of 50 nM. The Er-doped UCNP, NaLuF_4_:Yb,Er,Tm@NaLuF_4_, presented multiple emission peaks at 550 nm, 650 nm, and 800 nm, which were quenched by adding the PB shell. H_2_S triggered the decomposition of the PB shells to recover the strong upconversion luminescence (UCL) signal and the near infrared-to-green (N/G) ratio due to the cooperation of both redox and combination reactions. With the help of DL-propargylglycine (_DL_-PAG), the UC-PB was able to realize the *in vivo* near-infrared region ratiometric imaging, eliminate and inhibit the production of H_2_S, which is meaningful for clinical acute pancreatitis treatment.

### 2.3. Multi-Modal Signal

Fluorescence imaging (FLI), magnetic resonance imaging (MRI), and photoacoustic imaging (PAI) are widely used molecular imaging technologies which can realize noninvasive disease diagnosis and real-time *in vivo* lesion imaging [95,96]. Compared to single-modality imaging, which has the limitation of low penetration depth and low spatial resolution, multi-modality imaging integrates two or more modalities into one nanocomplex. Thus, it can provide more efficient and comprehensive information, which is desirable in the biomedical field [97].

Kuang and colleagues [53] developed an ultrasensitive and selective method for H_2_O_2_ detection based on a UCNP@ZIF-8/NiSx chiral complex. The NiSx moiety is a chiral nanoparticle with circular dichroism (CD) signals at 440 and 530 nm. The presence of the NiSx can quench the UCL signal of the UCNPs core at 540 nm, with the UCL signal at 660 nm remaining unchanged. The introduction of H_2_O_2_ caused NiSx to degrade, accompanied by the recovery of the UCL signal and the disappearance of the CD signal. This dual-mode signal of CD and fluorescence changes opens a new avenue for developing a toolbox for biomedical and biological analyses.

Yin and colleagues [98] reported a MnO_2_-coated, hollow mixed metal (Mn/Cu/Zn) MOF that could allow the photosensitizer, indocyanine green (ICG), to have a high loading efficiency. The coexistence of Cu^+^ and Cu^2+^ in the MOF, as verified by X-ray photoelectron spectroscopy, endows the complex with a glutathione-responsive “turn on” MRI ability. With the laser irradiation, the ICG can serve not only as the fluorescence and photothermal imaging agent, but also as the photodynamic therapy (PDT) agent. Therefore, this hollow MOF was used as a trimodality imaging-guided tumor therapy agent to highlight the efficiency of mixed-metal and mixed-valence strategies in tumor theranostic capacities.

For small functional molecule encapsulation, the risk of leakage and burst release is always a challenge [99]. To solve the aforementioned problem, Yang and colleagues [100] used the one-pot approach to prepare a Fe-based MOF (MIL-53) with defect structure due to the introduction of near-infrared dye (cypate). Further decoration of PEG and transferrin on the surface of nanoparticles (denoted as CMNP-Tf) was able to accelerate the passive and active targeting to the tumor region. The presence of cypate endowed the nanoparticles with excellent PAI and near-infrared fluorescence (NIRF) imaging properties, as well as reactive oxygen species (ROS) generation and photothermal therapy abilities. Furthermore, Fe also possesses a *T*_1_-weighted MRI contrast property. Therefore, the CMNP-Tf can realize the NIRF-, PAI-, and MRI-guided tumor targeting imaging-guided photothermal/photodynamic performance.

**Table 1:** Summary of MOF based fluorescence applications.

MOF	Synthesis Method	Luminescence Center	*λ*_ex/em_ (nm)	Target Molecules	LOD	Ref.
ZnMOF	solvothermal	TCPE		HCl vapor	2.63 ppm	[6]
CoMOF				histidine	2 × 10^−6^ M	
Eu-ZnMOF	solvothermal	Eu & BPDC	330/433 & 615	vanillylmandelic acid		[32]
TbTATAB	solvothermal	Tb & linker		Hg^2+^	4.4 nM	[39]
CDs@ZIF-8	one-pot room temperature crystal growth	CD	365/480	quercetin	3.5 nM	[40]
Ag_12_bpy-NH_2_		bpy-NH_2_	370	O_2_	11.4 mPa	[42]
Tb(III)@Cd-MOF	solvothermal	Tb^3+^	325/544	diphenyl phosphate	0.022 mg/mL	[47]
MIL-101(Cr)	hydrothermal	Cy3	525/570	tetrodotoxin	0.006 ng/mL	[60]
ZIF-8⊃4-MU & Flu	solvothermal	4-MU & Flu	360/380–450 & 500–570	temperature		[77]
Zr-MOF	Suzuki coupling	Zr-MOF-ABt Zr-MOF-BDP	340/403 & 530	peroxynitrite		[78]
			340/403 & 610			
R-UiO	Suzuki coupling	DBP-Pt/RITC	514/570 & 630	O_2_		[79]
EuMOF-FITC	hydrothermal	Eu^3+^ & FITC	380/525 & 611	biogenic amine	1.11 mg/L	[80]
Eu^3+^/Tb^3+^ MOFs	solvothermal	Eu & Tb	280/547 & 491, 616 & 592	Fe^3+^	3.86 μM	[83]
Eu_0.0069_Tb_0.9931_-DMBDC	solvothermal	Eu & Tb	355/613 & 545	temperature		[84]
Ru@MIL-101(Al)-NH_2_	one-pot	Ru & linker	300/465 & 615	water	0.02% *v*/*v*	[89]
UC-PB	ligand-exchange and controllable complexation	UCNP	980/540 & 654	H_2_S	50 nM	[94]
ZnMOF	solvothermal	Linker	370	Fe^3+^	28 μM	[101]
				Pb^2+^	600 μM	
				Cr_2_O_7_^2−^	43 μM	
				CrO_4_^2−^	45 μM	
CdMOF				Fe^3+^; Pb^2+^; Cr_2_O_7_^2−^; CrO_4_^2−^	57 μM; 370 μM; 71 μM; 31 μM	
ZJU-168(Tb or Eu)	solvothermal	Tb &linker	340/430 & 544	Glutamic acid	3.6 μM	[102]
		Eu & linker	340/430 & 614		4.3 μM	
F-UiO	solvothermal	FITC	488 & 435/520	pH		[103]
BSA + KFP@ZIF-8/HP +primer + MB	one-pot room temperature crystal growth	Cy5		survivin mRNA	2.3 pM	[104]

## 3. MOFs-Based Nanosensors in SERS Applications

Surface-enhanced Raman spectroscopy is a hypersensitive technique that enhances Raman scattering of the analyte in proximity to a nanostructured substrate via electromagnetic or chemical enhancement [4,105]. It can provide structural fingerprint information on the low-concentration analyte in real time [106,107]. Owing to its high sensitivity and high selectivity, SERS has a breadth of applications in pharmaceutical and environmental analysis [69,108], food science [109], life sciences [5], clinical diagnosis [64,69], and other fields [106]. However, for metal substrates, problems remain regarding the application of real-world sample detection. To name a few, the selective adsorption of analytes onto the metal substrate is required to assure better sensitivity and specificity [110]; the binding strength between the analyte and plasmonic surfaces needs to be improved to facilitate better chemical enhancement; and the stability and reproducibility of metal substrates need to be improved to assure the robustness of the analytical methods [105].

**Scheme 1:**
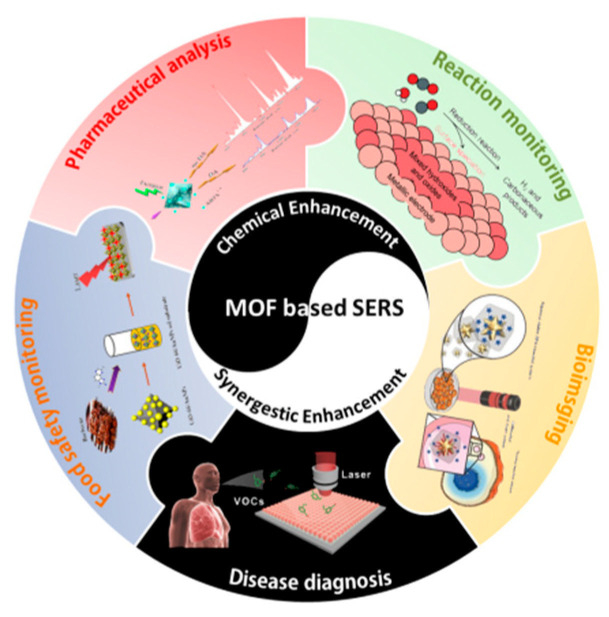
Applications of MOF-based surface-enhanced Raman spectroscopy (SERS).

As a new class of porous polymeric materials, MOFs present ultra-high porosity, a large surface area, and designable binding sites for multiple functionalization, which provide concentrating effects and multiple selectivity for the analytes [22,23,63]. In addition to directly serving as the SERS substrate, the MOF-metal composite SERS substrate can further improve both the sensitivity and the selectivity of the analytical measurements; in such a way, the stability and uniformity of the enhancement substrate can be improved [32,75]. Therefore, MOF materials have found increasing applications in SERS measurements (Scheme 1, Table 2).

### 3.1. SERS Substrates

To date, many MOFs have been directly used as SERS substrates; examples include ZIF-67, ZIF-8, Mo-MOF, MIL-100(Fe), etc. [91,112,114]. The enhancement by MOF can be attributed to chemical enhancement (CM) [63,105], which has been proposed as the primary enhancement mechanism for plasmon-free substrates such as MOF, semiconductors, and other metal oxides (Cu_2_O [73], WO_3_ [115], TiO_2_ [116], VO_2_ [117,118,119]), etc. Particularly, charge transfer transitions may be major contributors to SERS [120]. The charge transfers between the highest occupied molecular orbital (HOMO) of the analyte and the conduction band (CB) edge of the substrate material, or between the valence band (VB) of the substrate material and the lowest unoccupied molecular orbital (LUMO) of the molecules, are crucial for the enhancement effects. Other than the resonance Raman enhancement due to electronic transition between HOMO and LUMO, the electronic transition between VB and CB of the substrate material can also contribute to the enhancement of the Raman signal due to the resonance process [121,122,123].

The first MOF-enhanced Raman spectroscopic study was conducted by Tsung-Han Yu et al. [121] using methyl orange (MO) adsorbed in MIL-100 and MIL-101 as the model system. The study suggested that the Raman intensity enhancement was due to the charge transfer between the metal oxide clusters in MOFs and the adsorbed MO molecules. The study also suggested that the SERS effect was also orientation-dependent, which is in agreement with the basic understanding of Raman spectroscopy.

**Figure 5:**
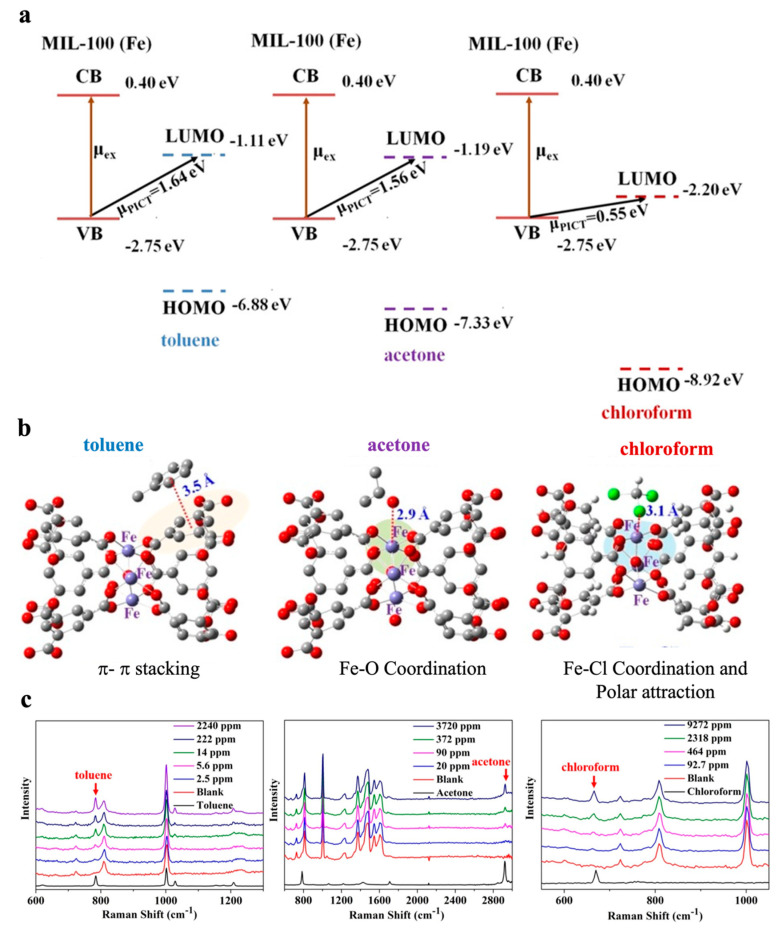
SERS of gaseous small molecules by MIL-100 (Fe). (**a**) Energy-level diagrams of toluene, acetone, and chloroform relative to MIL-100 (Fe) with respect to the vacuum level. (**b**) Adsorption affinity and binding energy of toluene, acetone, and chloroform on MIL-100(Fe). (**c**) SERS spectra of toluene, acetone, and chloroform vapor, respectively, on MIL-100(Fe), with concentrations as indicated [112]. Copyright 2020, John Wiley and Sons.

MIL-100(Fe) has been demonstrated, for the first time, by Li and colleagues to act as a SERS-active substrate to detect volatile organic compounds (VOCs) that usually possess low Raman cross-sections [112], and a LOD of 2.5 ppm was achieved for toluene. Based on special adsorption energy and density functional theory (DFT) calculations, the charge transfer enhancement mechanism was suggested for high SERS activity. Selective enhancement was attributed to resonance between laser energy and the photo-induced charge transfer energy, as well as the different dispersive energy between the ligand of the MOFs and the analyte (Figure 5). Through the ion exchange strategy, the MIL-100(Fe-Zr) complex improved LOD for isopropanol (50 ppm) compared to the pristine MIL-100(Fe) (100 ppm). This MIL-100(Fe)-based sensing platform was successfully used to monitor gaseous indicators, including 4-ethylbenzaldehyde, acetone, and isopropanol, for early diagnosis of lung cancer.

**Figure 6:**
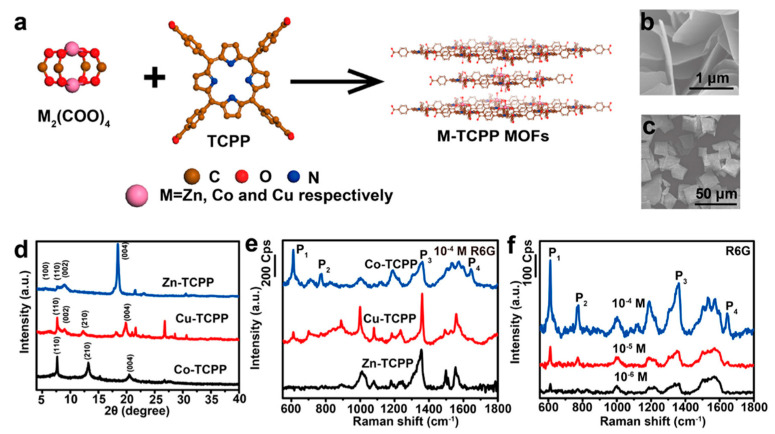
Structure and SERS effect of M-TCPP MOF. (**a**) Illustration of the process of synthesizing the M-TCPP (M = Zn, Co, and Cu) MOFs. (**b**,**c**) SEM images of the Cu-TCPP MOFs and Zn-TCPP MOFs. (**d**) PXRD patterns of the Co-TCPP MOFs, Zn-TCPP MOFs, and Cu-TCPP MOFs. (**e**) SERS spectra of R6G (10^−4^ M) on the Co-TCPP, Zn-TCPP, and Cu-TCPP substrates. (**f**) SERS spectra of R6G on the Co-TCPP substrate at three different concentrations: 10^−4^, 10^−5^, and 10^−6^ M [114]. Copyright 2019, American Chemical Society.

MOFs have a high degree of tailorability, with the ability to choose nodes and linkers as well as to tune the framework topologies. Consequently, the electronic energy band structure can be tuned to match the molecular orbital energy level of the analyte in order to facilitate controllable combinations of several resonances, such as the charge transfer, inter-band, and molecule resonances, in addition to the ground-state charge transfer interactions; thus, a significantly enhanced Raman signal can be achieved. Encouragingly, based on the pore-structure optimization and surface modification strategy, Zhao and colleagues synthesized a series of MOFs by using metal clusters M_2_(COO)_4_ (M = Zn, Co, and Cu) as the nodes and Tetrakis(4-carboxyphenyl)porphyrin (TCPP) or 2-methylimidazole as the linker (Figure 6) [114]. The electronic band structure of the MOFs can be tuned to match that of the target analyte, such that the enhancement factor (EF) of ZIF-67 can reach as high as 1.9 × 10^6^ with the LOD as low as 10^−8^ M. The high flexibility of the MOF structure can provide high levels of variety for SERS applications.

Using MOFs as the SERS-active substrate can realize selective enhancement of the target molecule for the Raman signal. However, precise tuning of the band energy is required to facilitate resonance with laser energy and charge transfer energies, which makes it difficult to realize SERS for a breadth of molecules of interest using one MOF substrate. Furthermore, there is still room to improve the sensitivity which can be achieved by the combination of MOFs with plasmonic substrates.

Electromagnetic enhancement (EM) is a physical enhancement process attributed to the localized surface plasmon resonance (LSPR) of the noble metal [4]. When the laser impinges on the metal nanostructure, e.g., a metal nanoparticle, the electromagnetic wave causes the collective oscillation of the delocalized conduction electrons. LSPR occurs when the frequency of the light matches with the oscillation frequency. The coupling of incident light with the metal nanostructures results in a huge enhancement of the local electromagnetic field, which is the major mechanism accounting for SERS. The combination of MOF with noble metal SERS substrates may be able to realize the synergetic enhancement by integrating CM and EM into one system, thus achieving maximum sensitivity [23,63]. Furthermore, such a MOF-SERS combination may be but one solution for problems associated with metal substrates, such as the low concentration and high mobility of the analyte at the enhancement spot, complicated matrix interferences, and modest substrate stability [63,124]. Specifically, the adjustable pore size of MOFs enables them to serve as molecular sieves to filter the analyte based on molecular size [71,75,104]; MOFs’ shells can protect the metal substrates from oxidation and reactive species in the complex matrix, thus improving the stability [125].

**Figure 7:**
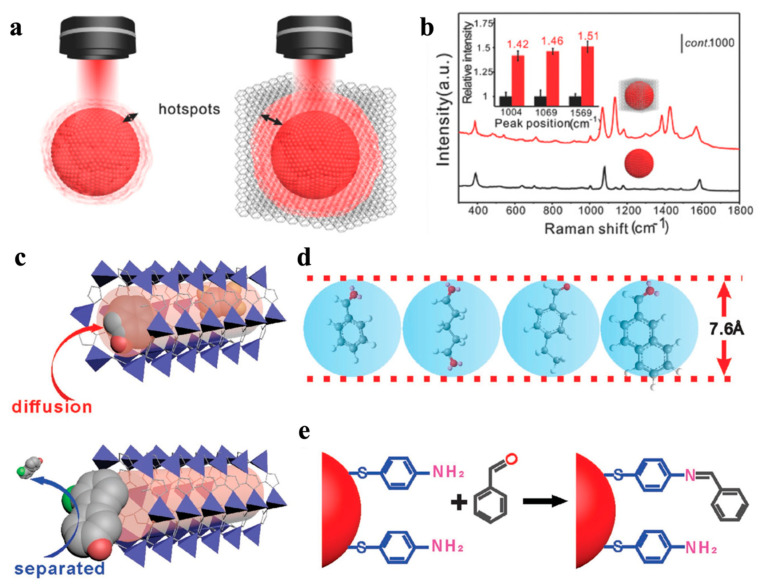
GSPs@ZIF-8 and the mechanism for selective SERS detection. (**a**) Hotspots in GSPs (left) and GSPs@ZIF-8 (right) around the edges of the plasmon. (**b**) SERS spectra of 4-ATP on GSPs (black) and GSPs@ZIF-8 (red), with the inset showing the relative intensity of the Raman spectra. (**c**) An illustration of the principle for achieving the selectivity. (**d**) The maximum diameter of four main analytes that were allowed to pass through the pores of ZIF-8. (**e**) The reaction used to capture aldehyde vapors via covalent linkage to the GSPs [64]. Copyright 2018, John Wiley and Sons.

MOFs coatings have the advantage of controlling the hotspot distribution to improve the SERS performance [71,72,73]. In the SERS technique, the Raman signal can be significantly enhanced only when the analyte is confined to the proximal distance to the plasmonic surface (<3–5 nm), because enhancement depends on the exponential decay of the plasmonic field at the metal–medium interface [63]. In the work by Wang and colleagues, gold superparticles (GSPs) were used to provide high-density hotspots, and the coating of ZIF-8 over GSPs provided a further enhanced SERS effect [64] (Figure 7). The finite-difference time-domain (FDTD) calculation revealed that the MOF coating was able to enhance the intensity of the electromagnetic field around the metal surface, and the high dielectric constant of MOF prevents the decay of the electromagnetic field along the radial direction. Consequently, a very intense SERS effect was observed [64,75].

**Figure 8:**
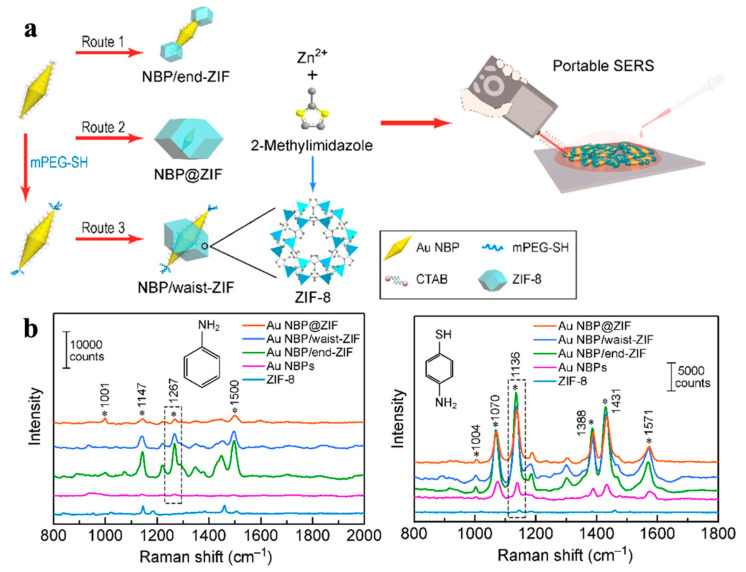
SERS of NBP@ZIF. (**a**) Schematics showing the routes for selective deposition of ZIF-8 on the Au NBPs and SERS detection. (**b**) SERS spectra measured with the ZIF-8 nanocrystals, Au NBPs, NBP/end-ZIF, NBP/waist-ZIF, and NBP@ZIF nanostructures for the detection of aniline and 4-ATP, respectively [71]. Copyright 2021, American Chemical Society.

The synergistic SERS effect was achieved by Wang and colleagues, via site-selective deposition of ZIF-8 on gold nanobipyramids (Au NBPs) [71]. Deposition of ZIF-8 was observed around the distal end, waist, or the surface of the Au NBPs (Figure 8). When ZIF-8 was located at the distal ends (Au BNPs@end-ZIF 8), the largest electric field enhancement was achieved, and the Raman signals on Au BNPs@end-ZIF 8 were at least twice those on the Au NBPs. Li and colleagues used ZIF-8-coated cuprous oxide/silica core–shell nanostructure (Cu_2_O@SiO_2_@ZIF-8) as a template to precisely control the growth of Ag NPs of varying sizes (2–29 nm) [73]. The results reflected that when the size of the Ag NPs matched well with the pores of Cu_2_O@SiO_2_@ZIF-8, the strongest electromagnetic field was generated. The Cu_2_O@SiO_2_@ZIF-8 provided abundant and uniformly distributed hotspots, thus resulting in an LOD of 5.76 × 10^−12^ mol⋅L^−1^ and a limit of quantification (LOQ) of 1.92 × 10^−12^ mol⋅L^−1^, respectively, in the detection of phenol red.

### 3.2. Other Effects of MOFs for Improving Selectivity and Sensitivity

Owing to the large specific surface area [20], uniform porosity [54], structural adaptability and flexibility [73,114], and ease of functionalization [63], MOFs as shells can enhance the selectivity and specificity of SERS substrates through physical adsorption and chemical recognition [72,110]. The introduction of additional functional layers, such as aptamers and antibodies, can ensure selective adsorption through multiple molecular interactions [23]. In addition, other factors, such as the thickness of MOFs, the nature of the metal species, and the functional groups afforded by the organic linkers [23,126], can also affect the selectivity.

The MOF shell over the plasmonic SERS substrate can serve as a sieve to allow only the target of interest to diffuse to hotspots, as well as to facilitate an efficient reaction with the Raman label by prolonging the contact time. VOCs are important biomarkers for early diagnoses of diseases [127], but the low concentration and high mobility of gaseous molecules result in insufficient collisions between gas molecules and SERS substrates, significantly compromising the detection sensitivity [128]. Wang and colleagues [64] used a GSPs@ZIF-8 SERS substrate to selectively detect aldehydes, a lung cancer biomarker in patients’ exhalation. The detection of gaseous aldehydes is currently limited by its small Raman cross section and poor adsorptivity on SERS substrates. The ZIF-8 shell allows the small vapor-phase aromatic compounds such as benzaldehyde, glutaricdiadehyde, and 4-ethylbenzaldehyde, but not 2-naphthaldehyde, to diffuse into the channel due to the sieving effect (Figure 7c–e). The prolonged contact time between gaseous molecules and the GSPs hotspots can facilitate the reaction of the analyte with the Raman-active probe molecule p-aminothiophenol (4-ATP) through the Schiff base reaction to generate a distinguished Raman signal. Apart from the physical adsorption, the MOFs can also selectively adsorb analytes through chemical recognition. Yusuke and colleagues [72] prepared hierarchical mesoporous Au films coated with homochiral MOF, which were able to realize ultrasensitive and enantioselective sensing of pseudoephedrine (PE) in complex bio-samples. The chiral ligands (a kind of alanine derivative) were used to endow the MOF with the homochiral property to distinguish (+)-PE. For analysis of PE in blood serum, the matrix effect was reduced by taking the advantage of the pore size limit to prevent large molecules from entering the MOF shell. This smart SERS substrate enabled an extremely low detection limit (10^−12^ M) in the complex biomatrix without preliminary separation.

In reality, the size of most of the biomarkers does not match that of the MOF channel. Efforts have been made to detect the metabolites as an indicator of the amount of biomarkers. By using an in situ reduction strategy, Zhou and colleagues [69] constructed a AuNPs@MIL-101@GOx (or AuNPs@MIL-101@LOx) nanoplatform for the detection of glucose/lactate, which are important neurochemicals associated with many physiological and pathological brain functions, such as ischemia, learning, and memory. The Raman-inactive reporter leucomalachite green (LMG) was oxidized into the active malachite green (MG) through a cascade of catalytic processes, and the signal intensity was used to indicate the amount of glucose/lactate. This nanoplatform has also been used to evaluate the therapeutic effects of astaxanthin for the purpose of alleviating cerebral ischemic injuries.

The metal-MOF-based SERS substrates have also been used in drug delivery and bioimaging due to the high surface area and porosity, excellent biocompatibility, and stability of MOFs [5]. The exposed active sites on the surface of the MOFs can be used for functionalization with recognition units, and the large specific surface area provides sufficient accessible binding sites for target molecules [80]. For example, the carboxyl group on the surface of the Au@Cu_3_(BTC)_2_ nanoparticles was used for functionalization with the aptamers [5]. In another example, the ZIF-8 shell on the Au@Ag surface was used to conjugate with IgG antibodies and recombinant nanobodies [70], based on the high affinity of polyhistidine toward transition metal ions [129]. The Au@Ag@ZIF-8 substrate was used to detect CD44 and EGFR biomarkers in mixed cell cultures, indicating the potential of the nanoprobes for SERS imaging and multiplexed bio-detection.

Farha and colleagues [113] reported the controlled encapsulation of gold nanorods (AuNRs) with a scu-topology Zr-MOF (NU-901) via the room-temperature assembly of MOF on AuNRs seeds. After incubating AuNR@NU-901 with a mixture of thiolated polystyrene (PST-SH; Mw = 5000 g/mol) and 4′-mercaptobiphenylcarbonitrile (BPTCN) molecules (roughly 15 × 7 Å along the thiol−CN axis and phenyl axis), the resulting spectrum closely matched with that of BPTCN alone. This result demonstrated that the prepared AuNR@MOFs were able to take-up molecules with suitable sizes and block large molecules from the pores, thus facilitating highly selective SERS detection at the AuNR ends.

### 3.3. Enhancement of the Stability, Homogeneity, and Reproducibility of SERS Substrates

The instability and reproducibility of the plasmonic nanoparticles under harsh environment represent inherent challenges in SERS detection [4,63,65,72]; therefore, a protective layer is necessary. Due to their excellent chemical and thermal stability [130], as well as their mechanical robustness [131], MOFs are an ideal candidate to act as a stabilizing layer. For example, Liang and colleagues [65] prepared a dense MIL-101(Cr) film on the rough titanium oxide foil via a secondary growth method, and then the Ag^+^ was reduced to Ag on the surface of the film to form the Ag@MIL-101(Cr) film SERS substrate. In such a way, the excellent SERS effect and the high reproducibility of the SERS substrate were achieved to realize the detection of nitrofurantoin (down to 10^−7^ M) without any complex subsequent procedures. Li and colleagues reported a highly sensitive and continuously stable 3D substrate (Cu_2_O@SiO_2_@ZIF-8@Ag) for SERS detection of phenol red with a LOD of 5.76 × 10^−12^ mol⋅L^−1^ and LOQ of 1.92 × 10^−12^ mol⋅L^−1^, and a high enhancement factor of 1.7 × 10^7^ was achieved even after 35 days [73].

**Figure 9:**
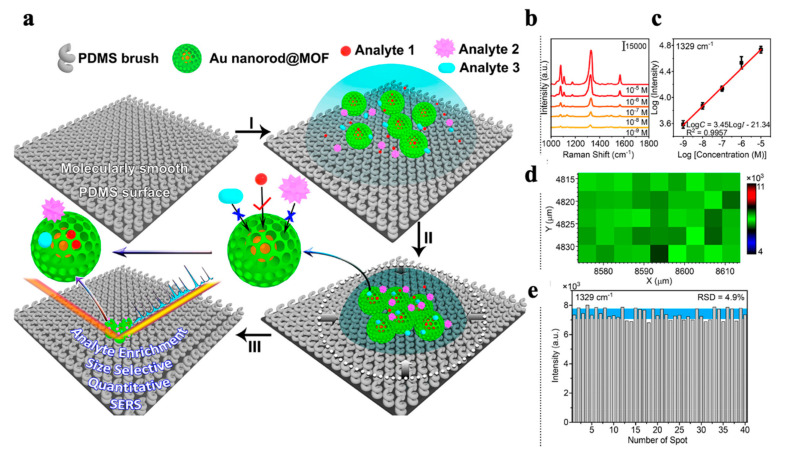
(**a**) Schematic illustration of the working principle of the AEF-SERS platform, showing application of the test solution (I), the enrichment and sieving effect (II), and the formation of analyte-SERS substrate aggregates (III) for selective and sensitive quantification of the analyte. (**b**) SERS spectra of 4-NBT at different concentrations using AuNR@ZIF-8 TRDs on the PDMS brush surface. (**c**) The calibration curve of 4-NBT at 1329 cm^−1^. (**d**) SERS mapping of 10 nM 4-NBT at 1329 cm^−1^ using AuNR@ZIF-8 TRDs. (**e**) Intensity variations at 1329 cm^−1^ from randomly chosen 40 SERS spectra using AuNR@ZIF-8 TRDs [75]. Copyright 2020, American Chemical Society.

The sensitivity and quantification performance of the SERS technique often contradict one another due to the modest reproducibility of the SERS substrate. Yang and colleagues constructed an integrated SERS platform with analyte enrichment and analyte filtration functions (referred to as AEF-SERS) to simultaneously achieve a good quantification performance and ultra-high sensitivity (Figure 9) [75]. In their work, single Au NRs were separated from each other through the coating of a thick ZIF-8 shell to form a AuNR@ZIF-8 submicroscale truncated rhombic dodecahedron (TRD); thus, a homogeneous SERS substrate was produced to improve the reproducibility of the detection. The separation of Au NRs may reduce the number of hotspots, thus compromising the sensitivity. However, the authors were still able to successfully realize a highly sensitive detection by constructing a polydimethylsiloxane (PDMS) brush surface that was capable of shrinking the analyte dispersion area by a million-fold in order to enrich the analyte.

The integration of noble metals and MOFs can speed up the development of the SERS technique. The high sensitivity can be partially explained by pre-concentration of the analyte through physical adsorption and chemical recognition. Aptamers, antibodies, and other recognition units can be easily used to modify MOFs in order to increase the molecular recognition specificity. In addition, when used in complex environment such as the biomatrix, the MOF shells provide a physical defense to improve the stability and reproducibility of the substrates, as well as to reduce the nonspecific adsorption and, thus, improving the detection sensitivity.

## 4. Conclusions and Future Perspectives

Applications of MOFs in the analytical and bioanalytical fields have experienced rapid growth due to their unique structural features. This mini-review summarized the advances of MOF-based optical detection methods, including luminescence and SERS, from the following aspects: the development of MOF-based luminophores, including the single luminophore signal, ratiometric signal and multi-modality signals; and the SERS effect of including MOFs as enhancement substrates and as auxiliary moieties for target molecule concentration, selective separation, and SERS substrate homogeneity for the purpose of improving the method’s robustness.

Compared to detection based on single parameter, the multiplexed detection with which multiple target or parameter detection is achieved in one sample volume can be more informational and can help to draw solid conclusions in the analysis of biological samples. Optical-based analytical methods have been used in the field of multiplexed detection due to their non-invasiveness, excellent spatiotemporal resolution, and, most importantly, their multiple coding elements, including intensity, wavelength, lifetime, location, and combinations of the above. Luminescent MOFs should be developed as an excellent type of multiplexing probe, because the broad choice of guest molecules and the structural diversity of MOFs provide diversified coding elements. However, the multiplexing capability of photoluminescent MOFs has been less frequently studied, if at all. Therefore, more efforts should be contributed to designing luminescent MOFs with multiple signal sources to facilitate the necessary analytical and bioanalytical applications.

Lanthanide ion-based MOFs, including mixed Ln ions, exhibit tunable luminescence peaks and lifetimes, making them suitable for ratiometric, multiplexed, and multi-modal measurements. However, as luminescent nanoprobes, the modest luminescence quantum yield in aqueous media impedes the application of these MOF-based luminescence sensors in aqueous solution as well as in biological samples. Future efforts should also include the engineering of the building elements of MOF structures to create more MOF-based optical nanosensors with improved performance in terms of factors such as physical and chemical stability, photostability, easiness in functionalization, quantum yield, red/near infrared emission wavelength, and tuned luminescence lifetime.

For the applications of MOFs in SERS measurements, the SERS mechanism of MOFs needs to be explored in more depth and breadth to rationally achieve the maximum SERS sensitivity. Further novel and facile approaches are expected to produce distinctive Raman signals via chemistry of the MOF with the analyte of interest. Efforts towards reproducible MOF substrates with high enhancement factors will be crucial to applications of MOF-based SERS in practical samples.

In both the photoluminescence and SERS fields, the breadth of applications need to be further explored in order to most effectively utilize the excellent physical and chemical properties of MOFs. The aforementioned needs represent challenges, but they also represent opportunities for MOF-based optical nanosensors to play a more significant role in bioanalytical applications.

## Figures and Tables

**Table 2:** Summary of MOF and MOF-plasmonic system-based SERS applications.

SERS Substrate	Target Molecules	Enhancement Mechanism	LOD	Ref
UiO-67	2,4,6-Trinitrophenol (TNP)	CM ^a^		[23]
Au/MOF-199	acetamiprid	EM ^b^ & CM	10^−8^ M	[42]
Au@MIL-101 (Cr)	tetrodotoxin	EM & CM	0.008 ng/mL	[60]
GSPs@ZIF-8	aldehyde VOCs	EM & CM	10^−9^ M	[64]
Ag@MIL-101 (Cr)	nitrofurantion	EM & CM	10^−7^ M	[65]
APTES@ZIF-67	benzaldehyde		10^−2^ M	[65]
ZIF-8	methyl orange		10^−4^ M	[65]
Au-L/D-AlaZnCl	pseudoephedrine	EM & CM	10^−12^ M	[72]
Cu_2_O@SiO_2_@ZIF-8@Ag	phenol red	EM & CM	10^−12^ M	[73]
Au/MOF-74	4-nitrothiophenol	EM & CM	69 nmol·L^−1^	[74]
Au@ZIF-8	4-nitrobenzenethiol	EM & CM	0.1 nM	[75]
Ag@MIL-101 (Fe)	dopamine	EM & CM	0.32 p M	[76]
Au/MIL-101	rhodamine 6G; benzadine	EM & CM	41.75 fmol; 0.54 fmol	[110]
Mo-MOF	crystal violet	CM	10^−6^ M	[111]
MIL-100 (Fe)	toluene	CM	2.5 ppm	[112]
	acetone		20 ppm	
	chloroform		20 ppm	
	isopropanol		100 ppm	
	4-ethylbenzaldehyde		26 ppm	
MIL-100 (Fe-Zr)	isopropanol		50 ppm	[112]
Au@MIL-101 (Fe)	toluene	EM & CM	0.48 ppb	[112]
Au@NU-901	4′-mercaptobiphenylcarbonitrile	EM & CM		[113]

^a^: Chemical enhancement; ^b^: electromagnetic enhancement.

## Data Availability

Not applicable.
